# Evaluating Psychological Effects of Amputation Through Virtual Reality Embodiment: A Study on Anxiety and Body Appreciation

**DOI:** 10.3390/jcm13237079

**Published:** 2024-11-23

**Authors:** Aina Manzano-Torra, Bruno Porras-Garcia, José Gutiérrez-Maldonado

**Affiliations:** 1 Bigfinite Inc., Carrer de Còrsega 301, 08008 Barcelona, Spain; 2 Department of Population Health Sciences, School of Medicine, University of Utah, 295 Chipeta Way, Room 1N490, Salt Lake City, UT 84108, USA; bruno.r.porras@hsc.utah.edu; 3 Department of Clinical Psychology and Psychobiology, University of Barcelona, Passeig de la Vall d’Hebron 171, 08035 Barcelona, Spain; jgutierrezm@ub.edu

**Keywords:** virtual reality, body appreciation, body anxiety, amputation, full body illusion, audio narration

## Abstract

**Background/Objectives**: A high number of patients who suffer the amputation of a lower limb will present psychological problems such as anxiety, depression, and post-traumatic stress disorder after surgery. This study embodies participants in a self-avatar with a right lower-limb amputation in a virtual reality environment. The aim was to determine if this experience increases anxiety levels compared to embodiment in a normal avatar. The study also examines whether body appreciation is related to anxiety levels. **Methods**: Subjects completed the Body Appreciation Scale (BAS) questionnaire before being immersed in the virtual environment, the Visual Analogue Scale for Anxiety (VAS-A) after each condition, and the Embodiment Questionnaire at the end of the experiment. **Results**: Univariate analysis showed that participants reported significantly higher levels of anxiety when exposed to the virtual avatar with an amputation compared to the full virtual body avatar. These results indicate that lower levels of body appreciation were associated with higher levels of anxiety across conditions, suggesting that participants with lower body appreciation experienced greater psychological maladjustment (measured by anxiety) in response to the virtual scenarios. **Conclusions**: The results suggest that the virtual avatar with a lower-limb amputation elicited significantly greater anxiety, and that body appreciation plays a key role in moderating this psychological response. Future research could focus on developing virtual exposure-based therapy for amputees using virtual reality to help reduce the anxiety experienced by patients during this process.

## 1. Introduction

Amputation is a surgical procedure involving the removal of a limb due to congenital, traumatic, or other causes [1]. In Catalonia, an estimated 1.78 per 1000 inhabitants live with amputation or the agenesis of a body part [2]. In Spain, the rate is 1.88 per 1000 inhabitants according to the same source. Globally, 57.7 million people were living with limb amputation due to traumatic causes in 2017, with the highest prevalences in East and South Asia, Western Europe, North Africa, North America, and Eastern Europe. Based on these data, approximately 75,850 prosthetists are estimated to be needed worldwide to care for patients with traumatic amputations [3]. The significance of amputations lies not only in their numbers, but in the profound changes and disruptions they bring to patients’ lives. Traumatic cases usually occur in young people, whose life projects and expectations are cut short [4]. The amputation of a limb not only results in the loss of a body part, but also affects the body’s capacities [5]. When a limb is lost, new needs, doubts, fears, and feelings such as inferiority, impotence, and uselessness appear, and the patient’s expectations and life projects are truncated.

According to biopsychosocial models, all personal dimensions are impacted during the amputation process, including cognitive, emotional, behavioral, and social aspects, which are influenced by prior experiences, beliefs, and thoughts [6]. The surgical procedure may also lead to mood and adjustment disorders, further deteriorating patients’ quality of life [1]. The same study emphasizes the importance of implementing psychological support before, during, and after the medical intervention, noting that psychological treatment effectively facilitates patients’ adaptation to their new condition by reducing anxiety and depression.

Participants, individuals who had had an amputation of a limb, shared the importance of viewing themselves in a large mirror to fully acknowledge the amputation as real [7]. Being able to look into mirrors is seen as a sign of acceptance of the amputation. However, patients may experience intense shock or distress the first time they see themselves in a mirror. This experience should ideally occur in a controlled setting, introduced by nurses, psychologists, or another amputee with a similar injury, rather than in a public scenario [7].

In the physical dimension, the main needs are those of mobility and acceptance of the physical state. This study’s results detail the acceptance of a body that has undergone a sudden alteration, the impact of which depends on the importance given to this factor by the patient [5]. In a study of the experiences of six army women who lost one or more limbs, researchers noted that all six women had difficulty adjusting to their mirror image. One participant stated that “At first, I wouldn’t look at myself in the mirror. Frightened to see how I really looked” [8] (p. 1446). In another study, a male amputee stated the following: “Seeing me without part of a leg was very hard…. I even needed support from the psychologist” [7,9] (p. 246). The Body Appreciation Scale (BAS) measures favorable attitudes toward one’s body, acceptance of imperfections, and rejections of unrealistic depictions in the media. A study involving Chinese college students found that women with higher scores on the BAS reported lower levels of anxiety and depression compared to their male counterparts [10]. Research using the Body Appreciation Scale (BAS) has consistently shown that higher body appreciation is associated with lower anxiety, particularly in the context of mirror exposure therapy. A study found that body appreciation, as measured by the BAS, increased after mirror exposure, especially in individuals with obesity, highlighting the role of this technique in promoting a healthier relationship with one’s body [11].

Mirror exposure therapy is a well-established technique to treat body-related anxiety. It is used in the treatment of different mental and physical disorders, such as anorexia nervosa, body dysmorphia, and phantom limb syndrome in the case of amputations. The technique involves patients confronting their own reflections to challenge negative body perceptions and develop body appreciation. For example, in eating disorders, mirror exposure is used to help patients move from a self-critical focus on body shape and weight to a more neutral or positive body image. Research shows that mirror exposure can promote improved body appreciation over time by reducing body dissatisfaction and anxiety [12,13]. In patients with obesity, mirror exposure has also been used to improve body image, guiding patients to focus on their body without judgment, leading to greater self-acceptance [12]. In cases of phantom limb syndrome, a mirror is used to create the illusion of a missing limb. It helps to reduce anxiety and pain by recalibrating the brain’s perception of the body [14]. This approach has been shown to improve body appreciation by restoring a sense of bodily integrity and control.

Virtual reality (VR) technology has recently been adapted to develop mirror exposure therapy. It offers an immersive platform on which individuals can be embodied in highly personalized avatars. VR has been used to treat patients with eating disorders, creating a space where patients can experience bodily changes in a gradual and supportive manner to encourage healthier body perception [15,16].

The present study is based on VR exposure. VR has also been frequently used in the treatment of phobias and post-traumatic stress, since it allows the person to be immersed in a controlled virtual environment, reducing the anxiety generated by the feared stimuli [17]. As VR is conducted in a controlled setting, it enables caregivers or experimenters to create artificial situations that closely resemble real life and to simulate participants’ bodies in 3D, which helps to generate positive results [18,19].

VR represents a new challenge for research in the medical field of amputations, where the majority of studies are related to phantom limb pain [20]. VR can decrease, or even eliminate, these pains or discomforts if the patient simulates moving the amputated limb within the virtual environment [20]. It is essential that the patient experiences the illusion of embodiment that appears when the participant feels that the avatar physically and functionally replaces his or her real body in the virtual environment [21]. The same authors define some aspects that can affect the elicitation of the embodiment illusion: ownership illusion, sense of agency and motor control, the location of the body, and the avatar’s external appearance. Ownership is based on the paradigm explained by the rubber hand illusion [22], but generalized throughout the body. In the rubber hand study, experimenters tactilely stimulate a visible rubber hand at the same time as the subject’s nonvisible actual hand. Subjects did not see their actual arm, but did feel the tactile stimulation synchronously and in spatial locations corresponding to the rubber arm.

A self-avatar is a collocated avatar that can replicate the user’s body posture and motions. This self-avatar is experienced from a first-person perspective and, within the VR, provides a substitute body for the participant [21].

Previous research indicates that patients who experience limb amputations often face significant emotional distress when they first see themselves in a mirror [23]. This initial shock is due to the psychological adjustment required to accept a new physical reality, which can lead to increased anxiety and discomfort when confronted with an altered body image. Mirror exposure therapy has been shown to alleviate some of this distress by promoting body acceptance and reducing anxiety over time [11]. However, the degree of anxiety triggered by mirror exposure, particularly in patients with amputations, remains a critical area of study.

Virtual reality (VR) presents a unique opportunity to extend mirror exposure into a controlled and immersive environment, allowing patients to confront their body image in a gradual and flexible manner [24]. VR has been widely used in the treatment of phobias and post-traumatic stress due to its ability to simulate real-world conditions while controlling anxiety-provoking stimuli. This feature makes it particularly suitable for addressing body image issues following amputation, where being confronted with one’s own altered appearance could trigger similar anxiety responses [25].

Therefore, the aim of the present study is to investigate whether a virtual avatar based on a self with a traumatic right lower-limb amputation can elicit a sense of embodiment and provoke emotional responses in limb-intact participants, similar to those experienced by real amputee patients. Specifically, we hypothesize that participants will experience higher levels of anxiety when exposed to a virtual body with an amputated right lower limb compared to a full-body avatar. This builds on previous findings that exposure to altered body conditions in a mirror can increase anxiety in the early stages of treatment [15], and that virtual reality offers an effective method to simulate and study these emotional responses in a controlled environment.

Body appreciation, which reflects positive attitudes and acceptance of one’s own body, has been identified as a key factor in predicting psychological responses to body-related stressors. The present research aims to explore whether levels of body appreciation can predict anxiety responses when individuals are exposed to stimuli related to their own body in a feared or distressing context, such as mirror exposure or virtual simulations of their bodies after amputation.

Therefore, the interest of the present research lies in studying the characteristics of patients before amputation in order to prevent psychological disturbance after surgery. More specifically, body appreciation is assessed as a potential predictor of subsequent psychological maladjustment. Body dissatisfaction occurs when an individual internalizes the culturally determined ideal body and, through social comparison, concludes that the perception of his or her body is incompatible with this ideal [26]. Understanding relevant factors could facilitate the allocation of resources for psychological treatment after amputation for patients.

## 2. Materials and Methods

### 2.1. Participants

The participant group was formed through a self-selection sampling method. A total of 36 volunteers (18 women and 18 men) from the Barcelona metropolitan area participated. The ages of the subjects ranged from 20 to 55 years (women: average age = 28.50, SD = 9.58; men: average age = 27.77, SD = 8.27). The volunteers were recruited through informative posters located in the Faculties of Psychology and Education at the Mundet campus and through advertisements on social networks. The experimental inclusion criterion was being between 18 and 60 years old. The exclusion criteria were having an amputated limb or first-degree relatives with amputations and the participant’s self-report of suffering from a major psychological disorder (for example, depression) or epilepsy.

### 2.2. Instruments

#### 2.2.1. Hardware and Software

Two different software applications were used for the avatars and virtual environment design. For the creation of the avatars (male and female) in 3D, Blender 2.78 v. (Blender Foundation, Amsterdam, Netherlands) was used, while the programming code necessary for the location of the avatars in the virtual environment was developed using Unity 3D 5.5 v (Unity Technologies, San Francisco, CA, USA). The virtual environment is a small room with white walls, one of which is a mirror. The avatar is located 1.5 m from the mirror, facing it. Avatars, both male and female, wear a basic white tank top, long black pants, and a hat to cover their hair and reduce possible misidentification with the virtual body. Avatars are designed specifically for each participant according to their body mass index (BMI) and by adjusting the silhouette. Using height and weight measurements, the virtual avatars are created to closely match each participant’s physical characteristics. It was required to take two photographs of each participant: one from the front with arms extended and legs slightly apart, and another from the side in the same posture. Using Unity software, the researcher adjusted the avatar’s dimensions to align with the participants’ silhouettes captured in the photographs, ensuring a realistic and personalized virtual representation.

The HMD HTC-Vive (HTC Corporation, Taoyuan City, Taiwan) was used to immerse the participants in the virtual world. In addition, three movement sensors and two HTC-Vive controllers were used, along with the headphones incorporated into the device, to achieve a more powerful immersive effect by synchronizing the movement of the image and the sound. The precise 360-degree tracking of the controllers and headset, realistic graphics, and directional audio translated into a realistic conception of the virtual body.

#### 2.2.2. Body Appreciation Scale (BAS)

The Body Appreciation Scale [27] consisted of 13 items that measured a single dimension to study the positive aspects of body image (favorable opinion in relation to one’s physical characteristics, body acceptance regardless of weight, shape, or imperfections, respect and attention to the body’s needs by adopting healthy behaviors, and self-protection by rejecting the ideals presented by the media) [28]. Each item was scored on a 5-point Likert scale ranging from 1 (never) to 5 (always). Higher scores indicated greater body appreciation. The scale was validated with a Spanish sample aged between 12 and 20 years, presenting adequate construct validity as well as adequate internal consistency, with a Cronbach’s Alpha of 0.91 [28]. The sample of the present study presented a Cronbach’s Alpha of 0.85. This scale has been used in numerous investigations related to positive body image [29].

#### 2.2.3. Visual Analogue Scale for Anxiety (VAS-A)

The VAS-A is a self-reporting instrument of subjective measures derived from patient experiences. The psychometric properties of the VAS-A were determined using different techniques, all of which proved to be adequate [30]. Validity was analyzed through concurrent and discriminant validity, while reliability was assessed with test–retest studies. The instrument consists of a 10 cm horizontal line with “not anxious at all” and “very anxious” marked on the left and right sides, respectively. The subject indicates the degree of anxiety experienced at that moment by marking a point on the line [31]. In the present study, it was used to measure the anxiety level felt by the participant during the experiment and was administered orally. The task was explained to the participant while they were wearing the VR HMD: “Imagine a line that goes from 0 to 100 where 0 is not anxious at all and 100 is very anxious. Indicate the value of the anxiety level you feel at this moment”.

### 2.3. Procedure

A within-subject design was employed, allowing each participant to serve as their own control to more precisely assess the impact of the amputation condition on anxiety. Initial anxiety levels were measured in the first condition, where participants were embodied in a complete avatar, using the VAS-A scale. That created a baseline to control the anxiety that could result from the immersive virtual reality experience itself, preventing it from influencing the findings. The BAS was used as a complementary measure to explore whether initial levels of body appreciation might predict anxiety levels during the second VAS-A measurement in the amputation condition. 

Before starting the experiment, the participants voluntarily signed a consent form that detailed the procedure and informed them about the confidentiality of their data as well as the possibility of leaving the experiment at any time.

**Figure 1:**
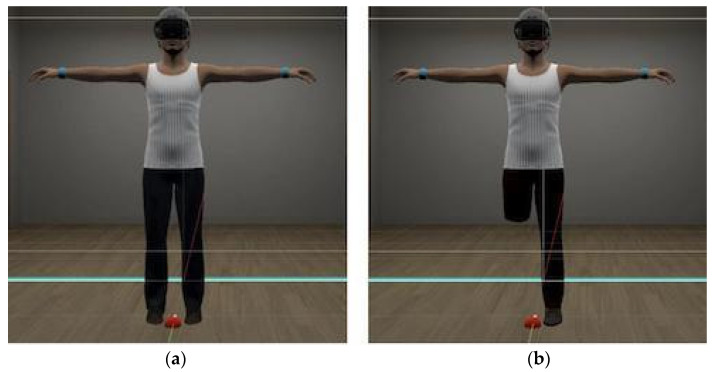
Avatars’ appearance: (**a**) real-body standard avatar; (**b**) lower-limb amputation standard avatar.

First, all participants completed the BAS questionnaire. Then, their virtual avatar was created. After that, they were embodied in a virtual body designed in the likeness of their own body (Figure 1). The embodiment was achieved through a visuo-tactile procedure, which was performed by applying physical contact from the HTC-Vive controller to the arms and legs (15 s each limb) of the participant while they observed both the first-person view and the mirror reflection. A visuo-motor stimulation procedure was also performed [32]. Visuo-tactile and visuo-motor stimulation were applied to enhance the illusion of embodiment, as explained in the introduction of this paper.

The experiment consisted of two conditions that all participants completed. Both took place in the same virtual room; the difference was that the avatar in the first scenario showed the whole body, while in the second scenario, it had the right lower limb amputated. The scene was a room with white walls. The avatar was positioned 1.5 m from the mirror, facing it. The participants kept the HMD on throughout the experiment.

In the first condition, the participants were embodied in a complete avatar. They were asked to explore their image in the mirror and in first person for 30 s. At the end of the first condition, anxiety was evaluated by orally asking the participants to rate their anxiety level on a scale from 0 (lowest level of anxiety) to 100 (highest level of anxiety) while looking at the virtual body. Afterward, the vision in the HMD was blocked. The participants kept the HMD on, and after a few seconds, the second condition began.

The second condition started with audio simulating a car accident, resulting in the amputation of the right lower limb of the avatar embodied by the participant. A few seconds after the audio finished, the participant regained vision in the HMD, and the room scenario returned, but now the avatar was missing its right lower limb (Figure 1). As in the first condition, participants needed to explore their virtual image in the mirror and in first person for 30 s. The anxiety level was then assessed, and the HMD was removed.

Finally, subjects answered the Embodiment Questionnaire [33]. This questionnaire sought to standardize the concept of embodiment in the context of VR. It consisted of six sub-scales that measured the different dimensions of the construct: sense of body ownership, sense of agency and motor control, tactile sensations, body location, external appearance, and response to external stimuli. The questionnaire had not yet been validated by itself, but comprised groups of the questions most often used in embodiment experiments from a review of 30 articles by [33].

## 3. Results

### 3.1. Statistical Analyses

The statistical analyses were carried out with the statistical program SPSS Statistics 26 (IBM Corp., Armonk, NY, USA) and Python 3.12.0 (Python Software Foundation, Beaverton, OR, USA). Prior to testing the main hypotheses, gender differences were assessed. Additionally, the study examined whether there was a correlation between age and the anxiety experienced in the second condition. Given the small sample size and the ordinal nature of the VAS-A and BAS data, non-parametric tests were selected. A Mann–Whitney U test was used to compare anxiety levels between male and female participants when embodied in the avatar with an amputated limb. Additionally, a Spearman’s Rho correlation was applied to determine any relationship between age and anxiety levels in the second condition.

A repeated measures ANCOVA was used to examine differences in anxiety levels between the two conditions, while controlling for body appreciation. The dependent variable was the score of the VAS-A under the two different conditions. The independent variable was the condition, which was a within-subject factor representing the different experimental conditions and had two levels: Condition 1 and Condition 2. Body appreciation was included as a covariate to control for its potential effect on the dependent variable and to account for individual differences in body appreciation that could impact the scoring of the participants. This approach allowed for a clearer understanding of the influence of the experimental conditions on the scores. The assumptions for ANCOVA were tested. Initial inspection revealed a violation of the normality assumption based on the Shapiro–Wilk test (*p* < 0.001), as well as visual deviations from the expected normal distribution in the Q-Q plot of residuals. To address this, a Box-Cox power transformation was applied to the dependent variable to normalize the data. After transformation, the normality assumption was reassessed and confirmed with the post-transformation Shapiro–Wilk test (W = 0.985, *p* = 0.552), and the Q-Q plot of the residuals indicated alignment with the theoretical normal distribution. Homogeneity of variance was examined using Levene’s test, which demonstrated no significant difference in variances across conditions (*p* = 0.908), supporting the assumption of homoscedasticity. Linearity between the covariate and dependent variables was verified through a scatterplot of residuals against the covariate, which displayed a random distribution around zero, indicating no systematic patterns or violations of the linearity assumption. Finally, no significant interaction was observed between the covariate and the independent variable, confirming the assumption of the homogeneity of the regression slopes.

### 3.2. Descriptive Results

The following results are expressed in relation to the entire sample, as there were no differences between men and women (U statistic = 209.5, *p* = 0.136) in terms of the anxiety reflected in the second condition, as indicated by a Mann–Whitney U test that did not reach the significance level (*p* < 0.05). The association between age and anxiety experienced with the avatar with the amputated limb, assessed by a Spearman’s Rho correlation, was not found to be significant (ρ = −0.129, *p* = 0.454).

First, the descriptive statistics of the total results of the questionnaires administered to the entire sample are presented (Table 1).

The repeated measures ANCOVA analysis (Table 2) was conducted to assess the impact of exposure to a full virtual body avatar (Condition 1) versus a virtual avatar with a right lower-limb amputation on anxiety levels, controlling for body appreciation as a covariate.

**Figure 2:**
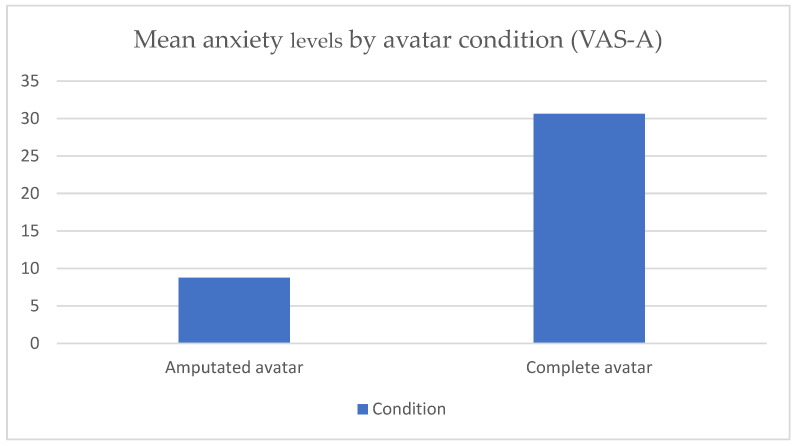
Bar chart comparing mean anxiety levels in the complete and amputated avatar conditions.

Supporting the first hypothesis, the analysis revealed a significant effect of condition on anxiety levels (F(1, 69) = 30.42, *p* < 0.001). Participants reported significantly higher levels of anxiety when exposed to the virtual avatar with a right lower-limb amputation compared to when exposed to the full virtual body avatar (Figure 2). This finding supports the hypothesis that exposure to an amputated virtual body would cause greater anxiety than exposure to a complete virtual body.

Regarding the second hypothesis, body appreciation was found to be a significant predictor of anxiety levels (F (1.69) = 12.80, *p* < 0.001). Lower levels of body appreciation were associated with higher levels of anxiety across conditions, suggesting that participants with lower body appreciation experienced greater psychological maladjustment (as measured by anxiety) in response to the virtual scenarios.

## 4. Discussion

As previously highlighted, traumatic amputations are not significant due to their prevalence in the population, but because of the abrupt alterations they cause in the lives of patients, affecting them physically, psychologically, and socially. An intense emotional response following a traumatic amputation is common among these patients and is a critical aspect of their psychological adjustment [34]. Long-term follow-up studies have shown that more than half of traumatic amputees develop formal psychological diagnoses, with the most common being post-traumatic stress disorder, anxiety, depression, and substance abuse [34].

The current investigation found no differences in the level of anxiety between men and women in the second condition, showing results consistent with previous studies. Most studies have found no differences between outcomes for men and women in terms of psychological well-being after amputation [35]. Despite mixed findings regarding the role of age, our study did not find significant differences in anxiety across age groups, although it remains an area worth exploring further, especially regarding the psychological expectations of younger amputees [23].

However, our results demonstrated a significant increase in anxiety levels when exposed to the avatar with amputation compared to the complete body avatar. Numerous studies reflect a diagnosis of anxiety after traumatic amputation surgery [25]. Trauma-based patients report shock, disbelief, and emotional numbness after amputation. The initial therapy phase includes exposure techniques such as viewing oneself in a mirror. They are conducted after the medical intervention and can bring intense anxiety and distress, particularly related to body image and physical safety, as patients struggle to integrate the loss into their self-perception [36,37]. Mirror therapy and supportive peer interactions may help patients accept their new body condition and build confidence in their physical capabilities [37]. Anxiety management is crucial at this time because it may precipitate other psychosocial factors such as unemployment, relationship breakdown, alcohol dependency, and drug abuse [35].

Additionally, the present study examined the relationship between pre-amputation body appreciation, as measured by the BAS questionnaire, and the anxiety experienced when participants viewed the amputated avatar. A negative correlation was observed between these two variables, suggesting that lower body appreciation prior to the amputation predicts higher anxiety during exposure to the virtual amputated body. This finding is consistent with research indicating that negative body image perceptions are closely associated with increased anxiety post-amputation [23,35]. This is particularly evident in younger patients, where the discrepancy between body ideals and reality can exacerbate emotional distress. The BAS score significantly influenced anxiety scores (*p* < 0.001. However, the main effect of the treatment condition remained significant, indicating that the VR amputation itself contributed to the anxiety increase, independent of baseline BAS. Future research could explore whether the BAS score may serve as a predictor of body image maladjustment following amputation.

VR has recently gained prominence as a tool to replace or enhance traditional mirror therapy. For example, in the case of amputees, it is used to help manage phantom limb pain and improve psychological outcomes [38]. VR creates controlled and ecologically valid environments that allow amputees to cope with their new body condition through interactive experiences [39]. It has also been studied for its ability to improve empathy in non-amputees by simulating the experience of having an amputated arm. This simulation enhances senses of embodiment and helps users better understand the challenges faced by amputees [40]. In the present study, VR exposure was used to simulate the experience of body amputation, allowing participants to confront their altered body image in a controlled setting. The use of VR in therapeutic contexts has been gaining attention, especially for its potential to address anxiety and trauma-related disorders. Previous research shows that VR can be effective in reducing anxiety by helping individuals gradually acclimate to feared stimuli in a safe and immersive space [41]. This method has proven successful in treating various anxiety disorders, including phobias, PTSD, and social anxiety [42], suggesting it may also benefit patients dealing with amputation. VR exposure offers the opportunity to repeatedly simulate the post-surgery experience without the immediate emotional shock of a physical confrontation, which could facilitate psychological adjustment [43].

The current findings suggest that anxiety levels increase when participants are first exposed to an avatar with an amputated limb, emphasizing the potential of VR as a preparatory tool to reduce anxiety before real-life exposure. Future applications could involve VR simulations in which patients with amputations are gradually introduced to virtual prostheses. This approach could help prevent rejection and facilitate the acceptance of prostheses while they are being manufactured [43]. Further research into integrating VR with psychological therapy could enhance emotional adjustment after traumatic amputation by minimizing the psychological impact of initial exposure to an altered body.

While this study provides valuable insights into the psychological impact of amputation and the potential of VR as a therapeutic tool, several limitations should be considered when interpreting the findings. First, the sample size of 36 participants, while sufficient to detect certain effects, is relatively small, which may impact the robustness and generalizability of the results. Additionally, the sample was drawn exclusively from the Barcelona metropolitan area, with volunteers primarily recruited from academic settings. This introduces potential social and geographic biases, limiting the applicability of the findings to more diverse populations. Furthermore, while no statistically significant differences were observed between genders or age groups, these results may be specific to the characteristics of the current sample. Future research should aim to replicate these findings with larger, more socially and geographically diverse populations to ensure broader applicability and to better explore gender- and age-related responses to VR-based interventions. Lastly, although this study did not examine potential right/left or limb-specific differences, future studies could expand on these factors to assess possible variations in psychological or physiological responses.

In the future, VR could be used with real patients to reduce anxiety during the first exposure to their new body condition, as VR exposure techniques have been shown to be effective in reducing anxiety in other pathologies. It would also be appropriate to standardize the evaluation of these dimensions, since currently, different instruments are used with results that are not comparable to each other.

## 5. Conclusions

This study shows that the anxiety described by the participants was significantly higher in the exposure to the avatar with the amputated limb compared to the real body avatar. This research has also shown that the lower the pre-amputation body appreciation, the greater the anxiety experienced by participants after the amputation. These results suggest that the virtual avatar with a right lower-limb amputation elicited significantly greater anxiety, and body appreciation plays a key role in moderating this psychological response.

The BAS [27] and VAS-A [30] were selected as key measures in this study due to their reliability in assessing body appreciation and anxiety levels, respectively. Using these validated tools aligns with standardized psychometric tools, which enhances the study’s rigor. It also supports comparability and replication in VR research.

Little is currently known about the prevalence of anxiety and depression in this group of people, especially in the long term [25]. Furthermore, most adaptations to amputation studies are cross-sectional and have used non-comparable measures [35], which makes it difficult to study this area.

For future lines of research, it would be advisable to focus on the factors related to the well-being disturbance of patients with amputated limbs to offer appropriate coping therapy for their needs. Psychological intervention is crucial in this group, since after surgery patients may develop psychological disorders that may have a chronic course. Conversely, the process of accepting and adapting to the new situation may be delayed or disturbed by certain biological, psychological, and/or social factors.

Virtual reality may be effective for exposure techniques in subjects with lower-limb amputation. VR might be a good resource to conduct awareness campaigns for family members or the general population.

## Figures and Tables

**Table 1:** Descriptive statistics of the results of the questionnaires.

	N	Range	Min	Max	Avg	Variability	Kurtosis	Error
Embodiment Total (without response scale)	36	2.00	−0.04	1.96	1.03	0.07	0.05	0.76
BAS Total	36	37	28	65	51.13	1.27	1.22	1.26
VAS-A 1 Total	36	95	0	95	8.77	3.25	12.30	3.42
VAS-A 2 Total	36	100	0	100	30.63	4.43	0	4.43
N	36							

Note: Body Appreciation Scale (BAS), Visual Analogue Scale for Anxiety (VAS-A), Min (minimum), Max (maximum), Average (Avg).

**Table 2:** Analysis of covariance (ANCOVA) with repeated measures for the effect of condition (complete vs. amputated avatar) and body appreciation on anxiety levels.

	Sum Sq	Df	F	PR (>F)
C (Condition)	57.617495	1.0	30.417829	5.661744 × 10^−7^
BAS Score	24.243403	1.0	12.798746	6.395585 × 10^−4^
VAS-A 1 Total	36	95	0	95
Residual	130.699900	69.0	NaN	NaN

## Data Availability

The datasets generated during and/or analyzed during the current study are available from the corresponding author on reasonable request.
